# Attitude, Knowledge, and Practices Regarding Prediabetes in Saudi Arabia: A Cross-Sectional Study on Family Medicine Residents

**DOI:** 10.7759/cureus.69300

**Published:** 2024-09-12

**Authors:** Faisal Aljehani

**Affiliations:** 1 Department of Internal Medicine, College of Medicine, University of Jeddah, Jeddah, SAU

**Keywords:** attitude, family medicine, knowledge, practices, prediabetes, residents, saudi arabia

## Abstract

Background

Individuals with prediabetes are at an increased risk of developing type 2 diabetes mellitus (T2DM) and cardiovascular diseases and mortality from any cause. The treatment and early detection of prediabetes and T2DM can aid in the prevention of future health complications. The aim of this research was to assess the attitude, knowledge, and practices of family medicine (FM) residents in Saudi Arabia regarding prediabetes.

Methodology

This is an online cross-sectional survey study that was conducted between March and May 2023. The study population consisted of family medicine residents who are currently practicing their profession in Saudi Arabia.

Results

A total of 101 family medicine residents participated in this study. The study participants showed a positive attitude toward prediabetes management with a mean score of 25.4 (standard deviation {SD}: 4.8) out of 30. The study participants showed a moderate level of knowledge regarding prediabetes management with a mean score of 7.0 (SD: 2.2) out of 12. The proportion of participants who were able to identify risk factors that might prompt them to screen for diabetes mellitus ranged from 47.5% (n = 48) to 96.0% (n = 97). The participants' agreement levels for questions that examined their attitude toward the importance of prediabetes screening ranged from 73.3% (n = 74) to 90.1% (n = 91). The most commonly agreed upon barrier was the patient's lack of motivation (n = 80, 79.2%). The most commonly agreed upon interventions that facilitate management and treatment improvements of prediabetes were more resources for patient education and increased access to the diabetes prevention program (DPP), an evidence-based lifestyle change program (n = 80, 79.2%). A body mass index (BMI) of ≥35 kg/m^2^ was the most commonly reported factor that increases the likelihood of prescribing metformin for a patient with prediabetes. The most commonly agreed upon barrier to prescribing metformin for prediabetes patients was that patients do not like taking medications (n = 67, 66.3%).

Conclusions

The study participants demonstrated an average level of understanding and a positive attitude regarding the management of prediabetes. A significant percentage of the participants demonstrated comprehension of risk factors associated with diabetes, as well as appropriate screening methods, with fasting blood glucose tests being of particular interest. It is recommended to enhance prediabetes management through heightened awareness and education concerning screening methodologies and lifestyle modifications.

## Introduction

Prediabetes is a medical condition in which blood sugar levels are higher than normal but not high enough to be classed as diabetes. This encompasses individuals who have impaired fasting glucose and glucose tolerance [1]. Research reveals that individuals who have prediabetes have an increased risk of experiencing problems associated with diabetes, including cardiovascular diseases, nephropathy, and neuropathy [1]. The high prevalence of prediabetes is a cause for concern as they are the potential pool of developing type 2 diabetes mellitus (T2DM) in the future. Existing literature indicates that lifestyle therapies targeting obesity and the lack of physical activity can diminish the risk of prediabetes progressing to type 2 diabetes mellitus by as much as 58.0% [2-4].

Primary care physicians (PCPs) have a vital role in preventing diabetes by identifying and effectively managing prediabetes. Assessing the extent of primary care physicians' understanding of prediabetes is crucial for effective screening programs and the implementation of appropriate management measures [5,6]. Nevertheless, there is a lack of information regarding the extent of primary care physicians' (PCPs) expertise, both in Saudi Arabia and elsewhere [7]. The majority of patients with prediabetes do not receive preventive therapy that is supported by scientific evidence from their PCPs [8,9]. Physicians face obstacles at a systemic level that impede their capacity to deliver optimal evidence-based practice. These barriers include factors such as performance metrics, cultural expectations, insurance reimbursement, and inadequate tools and staffing resources [10].

The Scientific Board of the Saudi Board for Family Medicine (SBFM) works in the Kingdom of Saudi Arabia (KSA) under the umbrella of the Saudi Commission for Health Specialties, thus supervising all postgraduate family medicine (FM) programs [11]. The FM residency training program offers supervised guided learning opportunities in ambulatory care and hospital-based medicine through a four-year, full-time, and supervised residency training program in FM. The rotations of the SBFM program curriculum are as follows: There is a six-week introductory course, and in the following years up to the end of residency year 3 (R3), trainees undergo different rotations in various specialties apart from a three-month rotation in family practice each year. The trainee shall spend all of the fourth year in FM practice. Research methodology and fieldwork rotation are to be taken at residency year 2 (R2). The course in community medicine is to be taken at R3 [11].

Saudi Arabian medical education has taken steps toward preparing physicians to handle chronic diseases such as prediabetes, although much more needs to be achieved, in comparison with other developed countries. Most Saudi family medicine programs are developed based on the competency-based curriculum decided by international standards and emphasize evidence-based medicine and public health strategies. However, the literature indicates that actual implementation in terms of prediabetes screening and management by general practitioners shows gaps [12]. A study found that most Saudi primary physicians have a lower level of knowledge about the risk factors of prediabetes and less than half apply competent evidence-based practice in clinical practice [12]. Other countries, such as the United States and the United Kingdom, incorporate more chronic disease management into their family medicine curricula, so their residents are better prepared with more specialized training in lifestyle interventions and the early detection of prediabetes ​in a multidisciplinary manner. This may be due to the comparative deficiency in the nation's continuing education programs and the less frequent use of clinical guidelines specific to prediabetes management, relating to less proactive care compared with Western healthcare systems [12]. Keck et al. [13] and Tseng et al. [5] suggested that due to a lack of knowledge among professionals (specifically family medicine healthcare professionals {HCP}), there was an inadequate identification of prediabetes and referrals to programs that were targeted at preventing diabetes. A systematic review by Teoh et al. demonstrated that the knowledge, attitude, and practice (KAP) levels of healthcare professionals (HCP) were generally low [14]. Currently, there has been no comprehensive study conducted in Saudi Arabia that focused on the KAP of family medicine residents. The aim of this study was to examine family medicine residents' attitudes, knowledge, and practices regarding prediabetes in Saudi Arabia. It is important to understand the KAP of family medicine residents toward prediabetes since they are primarily involved in the early detection, prevention, and management of the condition and, therefore, have a very significant influence on patient outcomes. In general, their all-inclusive approach to care puts them in a unique position to establish preventive measures and to educate their patients effectively.

## Materials and methods

Study design

This is an online cross-sectional survey study that was conducted between March and May 2023 on family medicine residents in Saudi Arabia.

Sampling procedure

The study sample for this investigation was chosen using the convenience sampling technique. The current study included physicians who met the predetermined criteria for inclusion and indicated their willingness to participate in the research. At the beginning of the questionnaire, the participants were given an informed consent form, which allowed them to choose whether to continue participating in the study or to withdraw from it. The research objectives were explained in their entirety to improve the participants' comprehension of the importance of their participation. The study's invitation letter highlighted the inclusion criteria for the study.

Study population and recruitment procedure

The inclusion criteria for this study were family medicine residents who are currently practicing their profession in Saudi Arabia. There are no limitations or constraints based on one's age or gender. Healthcare professionals with other specialty areas other than family medicine were excluded from the study. The survey uniform resource locator (URL) was shared on several social media platforms, such as Facebook, WhatsApp, Snapchat, and Twitter, in order to encourage greater participation and interaction.

Questionnaire tool

In this study, the questionnaire tool was adapted based on a previously used validated tool [5]. The questionnaire assessed the participants' demographic characteristics; knowledge and practices in the screening of diabetes; risk factors that would encourage you to screen for diabetes; tests they are going to order to screen for diabetes in those whom they think the test might be indicated; values that correspond with the lower and upper limits of the laboratory criteria for diabetes mellitus and prediabetes diagnosis, that is, the range; the value that corresponds with the American Diabetes Association (ADA) recommendations for lifestyle modification in people with the condition of prediabetes; the attitude toward the importance of prediabetes screening practices, knowledge, and beliefs in the treatment of prediabetes; treatment interventions recommended as a first-line method to manage a patient with prediabetes; interventions used as initial management for a patient with prediabetes; those interventions that facilitate the management and treatment of prediabetes; practice knowledge; and beliefs of prediabetes medication treatment and barriers to prescribing metformin for prediabetes therapy.

Piloting of the questionnaire tool

The questionnaire tool underwent evaluation and validation by healthcare professionals associated with the University of Jeddah. The participants were asked about the clarity and face validity of the questions, as well as any difficulty they experienced in understanding them. Additionally, the participants were asked to give comments on any questions they found inappropriate. In addition, a preliminary inquiry was carried out utilizing a restricted sample (20 participants) from the intended recipients to evaluate their understanding of the survey tool. They confirmed that the questionnaire items are clear and easy to understand.

Survey translation

The forward-backward translation technique was implemented in this study. This translation technique focused on the intended meaning of the questionnaire items not on word-by-word translation.

Ethical approval

This study was reviewed and approved by the Bioethics Committee of Scientific and Medical Research at the University of Jeddah, Jeddah, Saudi Arabia (reference number: HAP-02-J-094). All participants provided their consent before participating in the study.

Statistical analysis

The statistical analysis for this study was performed using the Statistical Package for Social Sciences software version 29 (IBM SPSS Statistics, Armonk, NY). Categorical variables were presented using frequencies and percentages. The normality of continuous variables was examined using histogram, skewness, and kurtosis measures. Continuous variables were presented using the mean and standard deviation (SD). Binary logistic regression analysis was conducted to identify the predictors of having a positive attitude and a higher level of knowledge regarding prediabetes. The dummy variable for the logistic regression model was defined as the mean score of the study participants. The findings of the regression analysis were presented as odds ratio (95% confidence interval). The significance level was assigned as a p-value of less than 0.05.

## Results

Participants' demographic characteristics

A total of 101 family medicine residents participated in this study. Females comprised more than half of them (n = 55, 54.5%). The majority of the participants reported that they work at the Ministry of Health of Saudi Arabia (n = 71, 70.3%). Around 20.8% (n = 21) of family medicine residents reported that they practice their profession in Riyadh. The demographic characteristics of the study participants are presented in Table 1.

**Table 1:** Participants' demographic characteristics

Variable	Frequency	Percentage
Gender		
Females	55	54.5%
Males	46	45.5%
Practice organization (multiple-answer question)		
Ministry of Health	71	70.3%
University clinic	13	12.9%
National Guard	8	7.9%
Military	8	7.9%
Current practice city		
Riyadh	21	20.8%
Dammam	12	11.9%
Al Madinah	12	11.9%
Makkah	10	9.9%
Others	46	45.5%

Diabetes screening knowledge and practices

Table 2 shows the participants' responses to questions that assessed their knowledge and practices regarding diabetes screening. The proportion of the participants able to identify risk factors that might prompt them to screen for diabetes mellitus ranged from 47.5% (n = 48) to 96.0% (n = 97). The vast majority of the study participants (n = 95, 94.1%) reported that they ordered a fasting blood glucose test to screen for diabetes in at-risk populations. The majority of the study participants (n = 83, 82.2%) identified the lower limits of the laboratory criteria for the diagnosis of diabetes mellitus as the fasting glucose level of 126 mg/dL or hemoglobin A1c (HbA1c) level of 6.5% (n = 66, 65.3%). The same proportion of the participants in this study could correctly state the upper and lower limits, that is, the range for prediabetes diagnosis laboratory criteria as fasting glucose from 100 to 125 mg/dL, 77.0%, or hemoglobin A1c from 5.7% to 6.4% (n = 61, 60.4%).

**Table 2:** Knowledge and practices regarding diabetes screening *Item used to examine the participants' knoweldge BMI, body mass index; US, United States

Variable	Frequency	Percentage
1. Which of the following are the risk factors that might prompt you to screen for diabetes? [5] (multiple-answer question)		
History of gestational diabetes*	97	96.0%
Age above 45 years*	93	92.1%
Family history of diabetes in a first-degree relative*	92	91.1%
Sedentary lifestyle*	85	84.2%
BMI above 25 kg/m^2^*	79	78.2%
Dyslipidemia*	78	77.2%
Hypertension*	75	74.3%
Heart disease*	50	49.5%
Smoking*	48	47.5%
2. In your practice, which tests do you order to screen for diabetes in at-risk populations? [5] (multiple-answer question)		
Fasting blood glucose*	95	94.1%
Hemoglobin A1c*	84	83.2%
Two-hour oral glucose tolerance test*	26	25.7%
Non-fasting blood glucose*	23	22.8%
3. Please circle the values that correspond to the lower limits of the laboratory criteria for diagnosing diabetes mellitus [5]		
a. Fasting glucose (mg/dL) (126 mg/dL)*	83	82.2%
b. Hemoglobin A1c (%) (6.5%)*	66	65.3%
4. Please circle the values that correspond to the upper and lower limits (range) of the laboratory criteria for diagnosing prediabetes [5]		
a. Fasting glucose (mg/dL) (100-125 mg/dL)*	78	77.0%
b. Hemoglobin A1c (%) (5.7%-6.4%)*	68	67.3%
5. Please circle the value that corresponds to the American Diabetes Association recommendations for lifestyle modification for patients with prediabetes [5]		
a. Minimum weight loss (percent of body weight) (5%)*	38	37.5%
b. Minimum physical activity (minutes per week) (150 minutes)*	61	60.4%
6. Which guidelines, if any, do you use for diabetes screening?		
American Diabetes Association	89	88.1%
US Task Force for Preventive Services	11	10.9%
None	1	1.0%

About 37.5% (n = 38) of the study participants were able to identify the American Diabetes Association's recommendations for lifestyle modification for prediabetes patients in terms of the minimum weight loss of 5% and 60.4% (n = 61) for the minimum physical activity of 150 minutes per week. The vast majority of the study participants (n = 89, 88.1%) reported that they use the American Diabetes Association guidelines to screen for diabetes mellitus.

Attitude toward the importance of prediabetes screening

Table 3 presents the participants' attitudes toward the importance of prediabetes screening. The participants' agreement levels for questions that examined their attitude toward the importance of prediabetes screening ranged from 73.3% (n = 74) to 90.1% (n = 91). The most commonly agreed upon statement was that lifestyle modification can reduce the risk of diabetes (n = 91, 90.1%). The least commonly agreed upon statement was that identifying prediabetes in their patients helps them determine if they need to treat hypertension more strictly (n = 74, 73.3%).

**Table 3:** Attitude toward the importance of prediabetes screening

How strongly do you agree or disagree with the following statements? [5]					
	Strongly disagree	Disagree	Neutral	Agree	Strongly agree
Identifying prediabetes in my patients is important for managing their health	3.0%	3.0%	5.0%	16.8%	72.3%
Identifying prediabetes in my patients helps me determine if I need to treat comorbid conditions such as hypertension more aggressively	4.0%	8.9%	13.9%	30.7%	42.6%
Identifying prediabetes in my patients helps me determine if I need to treat elevated blood sugars	5.0%	3.0%	12.9%	29.7%	49.5%
Patients with prediabetes progress to diabetes more quickly than those with normoglycemia	4.0%	0.0%	16.8%	37.6%	41.6%
Lifestyle modification can reduce the risk of diabetes in my patients with prediabetes	3.0%	2.0%	5.0%	26.7%	63.4%
Metformin can reduce the risk of diabetes in my patients with prediabetes	5.0%	1.0%	15.8%	35.6%	42.6%

Knowledge, practices, and beliefs regarding prediabetes management

Table 4 indicates the knowledge, practices, and beliefs of the participants in relation to the management of prediabetes. The vast majority of the study participants (n = 92, 91.0%) confirmed that providing counselling on diet changes and physical activity to lose weight is the best (recommended) initial management approach for a patient with prediabetes. Similarly, 95.0% (n = 96) of the study participants confirmed that providing counselling on diet changes and physical activity to lose weight is their initial management approach to a patient with prediabetes. Around one-third of the study participants (n = 38, 37.6%) reported that for patients they diagnosed with prediabetes, they would ask them to repeat laboratory work after one year. Around half of the study participants (n = 51, 50.5%) reported that for patients they diagnosed with prediabetes, they would ask them to return for follow-up after three months.

**Table 4:** Knowledge, practices, and beliefs regarding prediabetes management *Item used to examine the participants' knowledge

Variable	Frequency	Percentage
1. Which of the following is the best (recommended) initial management approach to a patient with prediabetes? [5]		
Provide counseling on diet changes and physical activity to lose weight*	92	91.0%
Refer the patient to a behavioral weight loss program	4	4.0%
Discuss starting the patient on metformin	4	4.0%
Refer the patient for bariatric surgery	1	1.0%
2. In your practice and with your current resources, what is your initial management approach to a patient with prediabetes? [5] (multiple-answer question)		
Provide counselling on diet changes and physical activity to lose weight	96	95.0%
Discuss starting the patient on metformin	62	61.4%
Refer the patient to a nutritionist	60	59.4%
Refer the patient to a behavioral weight loss program	47	46.5%
Refer the patient for bariatric surgery	13	12.9%
I do not consider prediabetes a condition that requires specific management	6	5.9%
3. In a patient of yours that you diagnose with prediabetes, when, if at all, would you have him/her repeat laboratory work? [5]		
Three months	38	37.6%
Six months	19	18.8%
One year*	38	37.6%
Two years	3	3.0%
No specific recommendation	3	3.0%
4. In a patient of yours with prediabetes, when, if at all, would you have him/her return for follow-up in your clinic? [5]		
Three months	51	50.5%
Six months	17	16.8%
One year	24	23.8%
Two years	5	5.0%
No specific recommendation	4	4.0%

Barriers to lifestyle modification for patients with prediabetes

**Figure 1:**
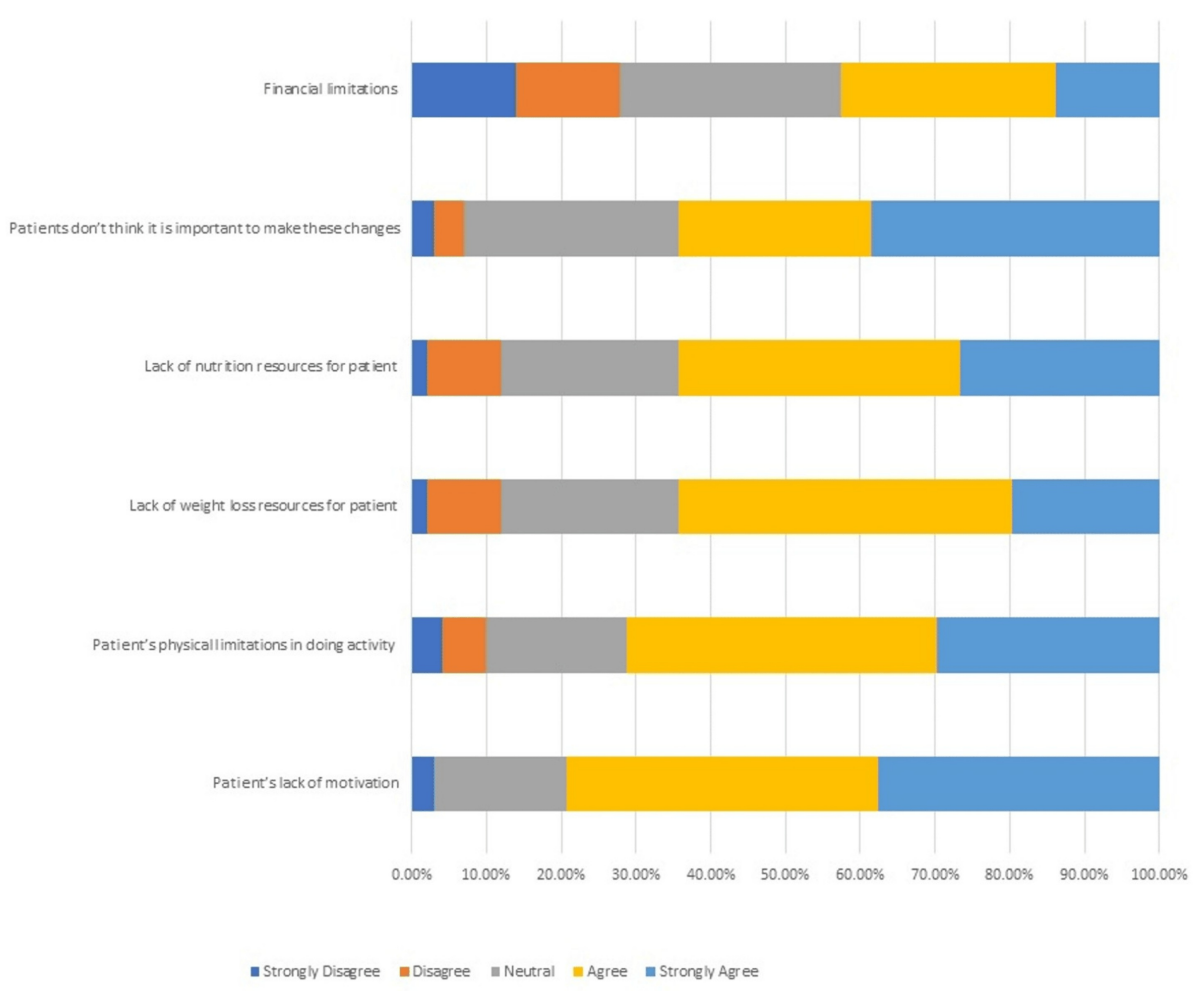
Barriers to lifestyle modifications

Figure 1 presents barriers to lifestyle modification for patients with prediabetes. The most commonly agreed upon barrier was the patient's lack of motivation (n = 80, 79.2%). The least commonly agreed upon barrier was the financial limitation (n = 43, 42.6%).

Management and treatment of prediabetes

Table 5 shows interventions that enhance the management and treatment of prediabetes. The interventions most agreed to that would enhance the management and treatment of prediabetes are more educational resources for patients and improved access to diabetes prevention programs (DPPs), evidence-based lifestyle change program (n = 80, 79.2%). The interventions that the respondents least agreed on as improving the management and treatment of prediabetes were improved access to bariatric surgery.

**Table 5:** Interventions that improve the management and treatment of prediabetes

How strongly do you feel that these interventions will improve the management and treatment of prediabetes? [5]					
	Strongly disagree	Disagree	Neutral	Agree	Strongly agree
More time for doctors to counsel patients	2.0%	2.0%	24.8%	39.6%	31.7%
More educational resources for patients	1.0%	1.0%	18.8%	36.6%	42.6%
Improved access to diabetes prevention programs (an evidence-based lifestyle change program)	2.0%	1.0%	17.8%	38.6%	40.6%
Improved nutrition resources for patients	2.0%	1.0%	20.8%	38.6%	37.6%
Improved access to weight loss programs	1.0%	2.0%	18.8%	36.6%	41.6%
Improved access to bariatric surgery	4.0%	8.9%	33.7%	28.7%	24.8%

Prediabetes treatment knowledge, practices, and beliefs

Table 6 presents the participants' knowledge, practices, and beliefs regarding the treatment of prediabetes with medication. A body mass index (BMI) of ≥35 kg/m^2^ was the most commonly reported factor that influences the prescribing of metformin for prediabetic patients. Around 29.7% (n = 30) of the study participants reported that they prescribed metformin for 6%-25% of their patients with prediabetes. The majority of the study participants (n = 72, 71.3%) confirmed that the guidelines for patients with prediabetes established by the American Diabetes Association are beneficial in the management of our patients.

**Table 6:** Knowledge, practices, and beliefs regarding the treatment of prediabetes with medication BMI, body mass index; HbA1c, hemoglobin A1c

Variable	Frequency	Percentage
1. Which of the following would make you more likely to prescribe metformin for a patient with prediabetes? [5] (multiple-answer question)		
BMI of ≥35 kg/m^2^	77	76.2%
History of heart disease	71	70.3%
Lack of response to lifestyle intervention	64	63.4%
History of gestational diabetes	53	52.5%
HbA1c of 6%	46	45.5%
Family history of diabetes	37	36.6%
Dyslipidemia	33	32.7%
Hypertension	28	27.7%
Age of <60	28	27.7%
I do not believe in prescribing metformin for patients with prediabetes	13	12.9%
2. Of your patients with prediabetes without progression to diabetes, what percentage of them have you prescribed metformin? [5]		
0%	11	10.9%
1%-5%	26	25.7%
>6%-25%	30	29.7%
>26%-50%	24	23.8%
>51%-75%	5	5.0%
>76%	5	5.0%
3. Have the American Diabetes Association guidelines for patients with prediabetes been helpful in managing your patients with prediabetes? [5]		
Yes	72	71.3%
No, I am not familiar with them	12	11.9%
Unsure	17	16.8%

Barriers to prescribing metformin for prediabetes patients

Table 7 presents barriers to prescribing metformin for prediabetes patients. The most commonly agreed upon barrier to prescribing metformin for prediabetes patients was that patients do not like taking medications (n = 67, 66.3%). The least commonly agreed upon barrier to prescribing metformin for prediabetes patients was the lack of US Food and Drug Administration (FDA) approval for metformin use in prediabetes (n = 18, 17.8%).

**Table 7:** Barriers to prescribing metformin for prediabetes patients FDA: Food and Drug Administration

How strongly do you agree or disagree that the following are barriers to prescribing metformin? [5]					
	Strongly disagree	Disagree	Neutral	Agree	Strongly agree
Patients do not like taking medications	2.0%	3.0%	28.7%	38.6%	27.7%
Medication cost to patient	11.9%	26.7%	37.6%	18.8%	5.0%
Poor patient adherence	2.0%	5.0%	33.7%	39.6%	19.8%
Potential side effects	4.0%	11.9%	34.7%	40.6%	8.9%
Providers' lack of awareness of clinical guidelines for metformin use	4.0%	13.9%	36.6%	38.6%	6.9%
Lack of FDA approval for metformin use in prediabetes	13.9%	26.7%	41.6%	15.8%	2.0%

Predictors of the participants' positive attitude and better knowledge

The study participants showed a positive attitude toward prediabetes management with a mean score of 25.4 (SD: 4.8) out of 30. The study participants showed a moderate level of knowledge regarding prediabetes management with a mean score of 7.0 (SD: 2.2) out of 12. Binary logistic regression analysis identified that there is no statistically significant difference in the participants' attitudes or knowledge based on their gender, practice settings, or practice city (p > 0.05) (Table 8).

**Table 8:** Predictors of the participants' knowledge

Variable	Odds ratio of having positive attitude (95% confidence interval)	P-value	Odds ratio of being knowledgeable of prediabetes management (95% confidence interval)	P-value
Gender				
Females (reference category)	1.00			
Males	0.84 (0.38-1.86)	0.675	1.11 (0.49-2.51)	0.801
Practice organization				
University clinic (reference category)	1.00			
Ministry of Health	1.00 (0.09-11.03)	1.00	0.24 (0.02-3.01)	0.268
National Guard	1.33 (0.31-5.68)	0.700	0.21 (0.03-1.84)	0.160
Military	1.33 (0.17-10.25)	0.782	0.19 (0.02-2.50)	0.207
Current practice city				
Riyadh (reference category)	1.00			
Dammam	2.20 (0.50-9.61)	0.295	0.86 (0.20-3.66)	0.840
Makkah	4.40 (0.75-25.84)	0.101	0.92 (0.20-4.31)	0.919
Al Madinah	1.10 (0.27-4.55)	0.895	6.77 (0.73-62.86)	0.093
Others	1.31 (0.47-3.68)	0.609	0.96 (0.33-2.77)	0.936

## Discussion

Diabetes mellitus is a complex metabolic disorder characterized by high blood glucose levels, often due to insulin deficiency or resistance [15,16], where it can lead to long-term damage and the dysfunction of various organs [17]. The management of diabetes requires diligent blood glucose monitoring, medication adjustments, adherence to a healthy lifestyle, and the treatment of comorbid conditions [18]. The importance of screening for diabetes is underscored by its impact on both physical and mental well-being [19]. Early detection is crucial, especially in light of the potential risks associated with intensive glycemic control [20]. Therefore, this study aimed to assess the knowledge, practices, and beliefs regarding prediabetes screening and management.

The study results found that between 47.5% and 96.0% of the participants were able to identify risk factors that might prompt them to screen for diabetes mellitus, where many studies have pinpointed several significant risk factors associated with diabetes mellitus, such as the lack of physical activity, obesity, smoking, advanced age, and a familial predisposition to the condition [21-23]. The vast majority of the study participants (94.1%) reported that they ordered fasting blood glucose tests to screen for diabetes in at-risk populations. In fact, the use of fasting plasma glucose measurements has been found to be an effective screening method, particularly for individuals with major risk factors for type 2 diabetes mellitus (T2DM) [23]. Moreover, the majority of the study participants (82.2%) identified correctly the lower limits of the laboratory criteria for diagnosing diabetes mellitus, where this is in line with the American Diabetes Association's (ADA) emphasis on fasting plasma glucose levels for diagnosis, with a cutoff of 126 mg/dL (7.0 mmol/L) [24]. Indeed, a similar proportion of the study participants was able to identify correctly the laboratory criteria (the upper and lower limits) for diagnosing prediabetes as fasting glucose levels of 100-125 mg/dL (77.0%) or hemoglobin A1c levels of 5.7%-6.4% (60.4%), where the position statement of the ADA defines prediabetes as a fasting plasma glucose level falling within the range of 100-125 mg/dL (5.6-6.9 mmol/L) [25]. A study conducted in the Al-Hasa region of Saudi Arabia found that PCPs showed significant deficiencies in their understanding of diagnosing, screening, and managing prediabetes [26]. A separate study conducted in the Al-Qassim region found that PCPs had inadequate knowledge, attitudes, and behaviors [27].

Approximately 37.5% of the participants in the study successfully recognized the ADA's recommendations for lifestyle modifications for individuals with prediabetes, that is, a minimum weight loss of 5%, while 60.4% identified the recommended minimum physical activity of 150 minutes per week, as the ADA's recommendations for lifestyle modifications for individuals with prediabetes included weight control, diet modification, and increased physical activity [28,29]. These recommendations are supported by studies showing that receiving healthcare provider advice is associated with improved adherence to these behaviors [29,30]. In fact, the statement most commonly agreed upon by the participants was that lifestyle modifications can decrease the risk of diabetes in individuals with prediabetes, with a consensus of 90.1%, where lifestyle modifications, including dietary changes, weight loss, and increased physical activity, have been shown to have a substantial impact on reducing the risk of diabetes. Interventions in the form of these lifestyle adjustments have proven effective in decreasing the progression from impaired glucose tolerance to type 2 diabetes, as evidenced by research [31,32].

Additionally, the vast majority of the study participants (91.0%) confirmed that providing counselling on diet changes and physical activity to lose weight is the best (recommended) initial management approach for a patient with prediabetes. Similarly, 95.0% of the study participants confirmed that providing counselling on diet changes and physical activity to lose weight is their initial management approach to a patient with prediabetes, where it was reported that healthcare provider recommendations and health coaching interventions were associated with improved adherence to healthy behaviors and significant reductions in hemoglobin A1c and weight [29,33].

Approximately, one-third of the participants in the study (37.6%) indicated that for patients diagnosed with prediabetes, they would recommend repeating laboratory tests after one year, where this is unlike the recommendations of the Australian Diabetes Society and the Australian Diabetes Educators Association for individuals with prediabetes, where routine testing of capillary blood glucose, hemoglobin A1c (HbA1c) levels, serum insulin, or pancreatic C-peptide levels, as well as testing for ischemic heart disease or microvascular complications of diabetes, is not considered necessary [34], as some studies suggest that most patients remained in the prediabetic range after three years [35].

The most commonly agreed upon barrier to lifestyle modification for patients with prediabetes was the patient's lack of motivation (79.2%), where this is in line with a range of studies that have identified the lack of motivation as a significant barrier to lifestyle modification for patients with prediabetes and type 2 diabetes [36-38]. The interventions most widely agreed upon as effective for enhancing the management and treatment of prediabetes included the provision of more educational resources for patients and improved access to DPPs, which are evidence-based lifestyle change programs, with a consensus of 79.2%; the importance of targeted prediabetes detection and improved accessibility to lifestyle intervention programs is underscored [39], with an emphasis on the effectiveness of DPPs when delivered by trained diabetes educators in reducing risk factors for diabetes and cardiovascular disease [30,40,41], as this standpoint is further supported by the development of an educational toolkit designed for use by health professionals and their patients with prediabetes [42].

The management of prediabetes involves reducing cardiovascular risk factors and intensive lifestyle intervention, with no role for routine testing [34], where a pathophysiology-based approach, including metformin and lifestyle therapy, can prevent progression to type 2 diabetes [43]. The study results found that the factor most frequently reported, making the participants more likely to prescribe metformin for a patient with prediabetes, was a body mass index (BMI) of ≥35 kg/m². In fact, patients with prediabetes, particularly those aged 60 years and older, with a BMI exceeding 35 kg/m², or with a history of gestational diabetes, are recommended to consider metformin as part of their management plan [44]. However, it was found that metformin initiation rates in patients with prediabetes are not in accordance with current recommendations [45], where around 29.7% of the study participants reported that they prescribed metformin for 6%-25% of their patients with prediabetes (without progression to diabetes), as most patients with prediabetes remain in this state, with or without intervention, and metformin use does not significantly impact glycemic outcome [35]. On the other hand, the barrier most commonly agreed upon, with a consensus of 66.3%, for prescribing metformin to patients with prediabetes was that patients do not like taking medications; indeed, patients' reluctance to take medications is a complex issue influenced by various factors [46], where for metformin, patients may also experience physical and psychological difficulties with large tablets and gastrointestinal disturbances, leading to poor adherence [47].

Based on the study's findings, it is recommended to enhance prediabetes management through increased awareness and education on screening practices and lifestyle modifications. The study reveals that a significant portion of the participants demonstrated awareness of diabetes risk factors and appropriate screening methods, with a focus on fasting blood glucose tests. Moreover, the majority correctly identified diagnostic criteria for prediabetes and recommended lifestyle modifications such as weight loss and increased physical activity. To address barriers, particularly patient motivation, it is advisable to implement targeted educational resources and improve access to DPPs. Additionally, there is a need to align prescribing practices with current guidelines, emphasizing the consideration of metformin for patients with a BMI of ≥35 kg/m². However, efforts should be made to address patient reluctance to medication through patient education and alternative formulations.

This study has limitations. The online survey study design and convenience sampling might have affected the generalizability of the study findings as the study population is restricted to users of social media platforms. Those who practice more or have stronger prediabetes opinions may be overrepresented. Random sampling or stratified sampling would have been preferable to ensure a more representative sample. In addition, the cross-sectional study design is not able to examine causality among independent and dependent variables. While the study collects valuable data on attitudes, knowledge, and practices, it cannot establish whether gaps in these areas are due to inadequate education, systemic barriers, or other factors. A longitudinal study might have been more informative in tracking changes over time and assessing the impact of educational interventions. Another limitation is the small sample size for a country-wide assessment. A large sample size would allow more robust conclusions and stronger generalizability. Furthermore, the absence of information about the ratio of invited family medicine residents to those who actually completed the survey (response rate) hinders the evaluation of the sample's representativeness. Additionally, the study's reliance on self-reported data introduces the possibility of response bias, as the participants may overestimate their knowledge or adherence to best practices. Therefore, the study findings should be interpreted carefully.

## Conclusions

The participants in the study exhibited a moderate level of knowledge and a positive attitude toward prediabetes management. A considerable proportion of the participants exhibited knowledge regarding risk factors for diabetes and suitable screening techniques, particularly fasting blood glucose tests. It is advisable to augment prediabetes management by means of increased awareness and training regarding screening methodologies and adjustments to one's lifestyle. Besides, there are clear gaps, particularly in understanding lifestyle modifications and follow-up strategies for prediabetes. These findings suggest that further efforts are needed to enhance both theoretical knowledge and its application in clinical settings.
